# Making Ends Meet: Repairing Breaks in Bacterial DNA by Non-Homologous End-Joining

**DOI:** 10.1371/journal.pgen.0020008

**Published:** 2006-02-24

**Authors:** Richard Bowater, Aidan J Doherty

## Abstract

DNA double-strand breaks (DSBs) are one of the most dangerous forms of DNA lesion that can result in genomic instability and cell death. Therefore cells have developed elaborate DSB-repair pathways to maintain the integrity of genomic DNA. There are two major pathways for the repair of DSBs in eukaryotes: homologous recombination and non-homologous end-joining (NHEJ). Until very recently, the NHEJ pathway had been thought to be restricted to the eukarya. However, an evolutionarily related NHEJ apparatus has now been identified and characterized in the prokarya. Here we review the recent discoveries concerning bacterial NHEJ and discuss the possible origins of this repair system. We also examine the insights gained from the recent cellular and biochemical studies of this DSB-repair process and discuss the possible cellular roles of an NHEJ pathway in the life-cycle of prokaryotes and phages.

## Introduction

Non-homologous end-joining (NHEJ) was first discovered in the early 1980s as a rather mysterious mechanism involved in the integration of certain viruses into mammalian genomes [1–3]. Initially described as “illegitimate” or “indiscriminate” recombination, it soon became clear that this process could join together any DNA ends [2]. The term “non-homologous” came into vogue once it was realized that there was little or no homology between the DNA being incorporated and its site of integration, making this pathway distinct from the more familiar process of homologous recombination (HR) [4–7]. To illustrate the fact that the pathway is completely distinct from HR, the process has been called non-homologous “end-joining” since the mid-1990s [8–10].

It is now accepted that the NHEJ pathway acts to join breaks in genomic DNA, specifically double-stranded breaks (DSBs) [11–15]. This type of break may be introduced during normal physiological processes such as V(D)J joining of immunoglobulin genes, or may be due to errors in DNA metabolism (e.g., collapsed replication forks) or to exogenous damage (e.g., ionizing radiation). Thus, genomic DNA is frequently converted into DSBs, and efficient mechanisms are therefore required to deal with all types of breaks. Both NHEJ and HR can be utilized to repair DSBs, and eukaryotic cells use them to different extents. HR predominates in lower eukaryotes such as *Saccharomyces cerevisiae* [15]. In contrast, NHEJ appears to predominate in higher eukaryotes, probably as this pathway is active throughout the cell-cycle [12,14], whereas HR is limited to the late S and G2. NHEJ is perfectly suited to handling DSBs, as this pathway displays no sequence requirements for the recognition of DNA ends. This property is important when joining DSBs, as broken ends can have diverse chemical and physical characteristics.

**Figure 1:**
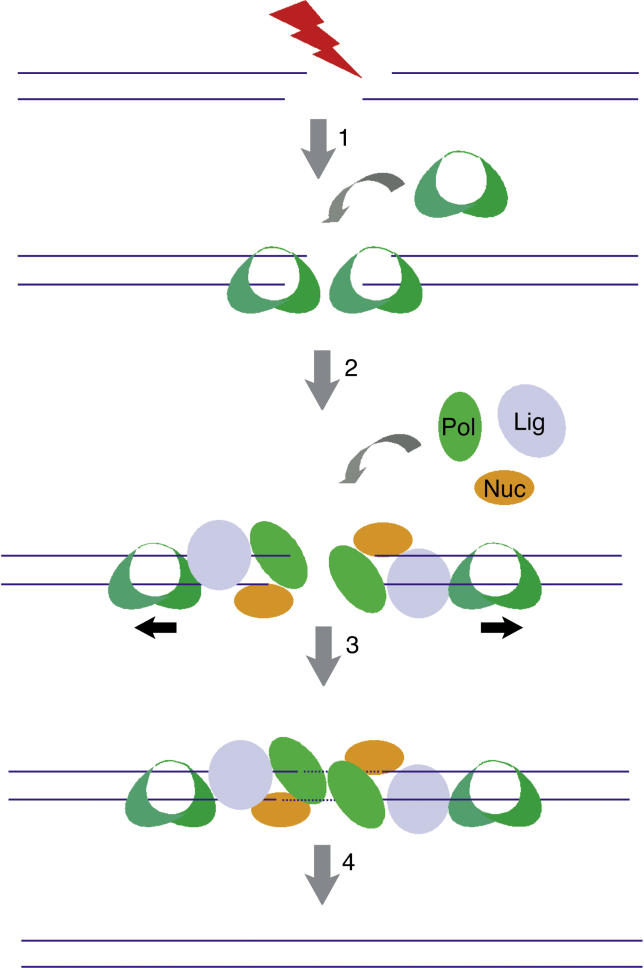
DSB Repair by the NHEJ Complex The Ku complex locates to the break site (step 1), where it may serve as an end-bridging and alignment factor. Following binding to the broken DNA ends, additional processing enzymes are recruited by Ku to the break site (step 2). Ku may translocate away from the ends, allowing access by other factors to the break termini. When DNA ends are non-complementary and/or are damaged, the DNA end-processing, gap-filling, and nucleolytic activities generate DNA termini, capable of being ligated, prior to ligation (step 3). Subsequently, the broken ends are joined by an NHEJ-specific DNA ligase (step 4) and the NHEJ complex dissociates.

The molecular details of eukaryotic NHEJ and its various cellular functions have been summarized comprehensively elsewhere [12,13,15]. NHEJ uses a variety of proteins that process the ends and join them together (Figure 1). To summarize, once a DSB has been created, the first step in the re-joining process involves the binding of the Ku complex to the two DNA ends. The next proteins to be recruited to the complex are those that lead to resection of the ends of the DSB. In vertebrates, these include the nuclease Artemis and the polymerases pol μ and λ. The final set of steps in NHEJ lead to direct joining of the two ends [16,17] by a specific DNA ligase that restores the integrity of the DNA [18–21]. Currently, it is not clear how the repair complex disassembles from the ligated DNA. Whilst most of the proteins can leave the repair site by passive dissociation, the Ku dimer is likely to be left encircling the DNA [22]. It has been suggested that proteolytic cleavage may be the only way to remove the Ku dimer from DNA [22–24].

Recently, homologues of several of the proteins involved in NHEJ have been identified in prokaryotes. Although the function of NHEJ in bacteria is still rather obscure, it now seems clear that this mechanism provides important physiological functions, at least in some bacteria. It has been hypothesized that such functions could include specialized forms of genome repair under conditions that lead to the accumulation of DSBs, or it may promote genetic diversity, particularly under difficult growth conditions where specific selection pressures may be in place. Here, we summarize current data and reflect on what these observations mean in terms of the evolution of the pathway and its function in different microorganisms.

## NHEJ in Prokaryotes

**Figure 2:**
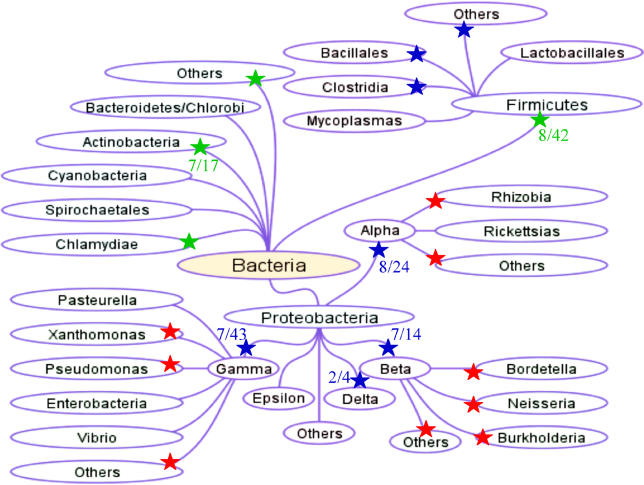
Phylogenetic Distribution of the NHEJ Ku Protein in Bacteria A diagrammatic tree representation of major phyla, classes, and families of bacteria, as indicated by the relevant names is shown. Lines between the names illustrate well-established phylogenetic links within the various phyla. Stars in green indicate phyla that contain homologues of Ku. Numbers in green indicate the number of species with completed genome sequences that are within each phylum and contain one (or more) homologue(s) of Ku. Stars in blue indicate classes/orders that contain homologues of Ku. Numbers in blue indicate the number of species with completed genome sequences that are within each class that contain one (or more) homologue(s) of Ku. Stars in red indicate families that contain homologues of Ku. Note that there is no instance in which a Ku homologue is contained in all species within a phylum/class.

**Discovery of Ku in prokaryotes and viruses.** Historically speaking, all of the major DNA-repair pathways were first described in prokaryotes and, subsequently, equivalent eukaryotic counterparts were characterized. As discussed above, the NHEJ pathway was initially discovered in the eukarya and only recently has a homologous NHEJ apparatus been identified in prokaryotes [25–29]. The phylogenetic distribution of Ku genes in different bacterial species is summarized in Figure 2. These genes are most widely distributed in proteobacteria (α, β, γ, and δ families), firmicutes, and actinobacteria (Figure 2). However, it should be noted that Ku genes are not present in all bacteria and are conspicuously absent from many widely studied bacterial strains, such as *Escherichia coli* K12 (enterobacteria). The specific factors that link organisms possessing NHEJ proteins have yet to be identified. There is no obvious phylogenetic pattern between the bacteria that possess and those that do not possess this apparatus, leading to the possibility that NHEJ systems may most commonly be acquired by horizontal gene-transfer events. This important issue will be discussed later in the review.

**Figure 3:**
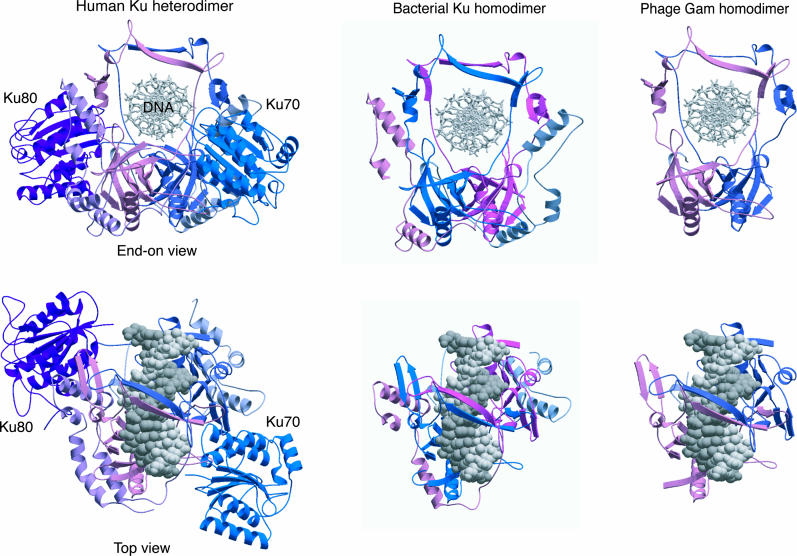
Structure of Eukaryotic, Prokaryotic, and Phage Ku Proteins The structure of the human Ku heterodimer bound to DNA [22] is depicted in two different orientations. Ku encircles the end of the DNA like a “nut on a bolt.” The predicted structures of the Ku from Mt and Gam homodimers are also shown. The smaller Ku homologues retain the regions required for dimerization and DNA binding but lack the N-terminal vWA domains, the C-terminal SAP (Ku70), and the DNA-PKcs binding (Ku80) domains present in the larger eukaryotic Ku proteins.

Ku is a major marker for the presence of the NHEJ pathway in many organisms, and the discovery of Ku homologues in prokaryotes led to the discovery of an NHEJ apparatus in many bacterial species [26,27]. The bacterial Ku proteins are approximately 30–40 kDa in size, in contrast to the much larger eukaryotic Ku complexes (70–80 kDa). The smaller bacterial Ku homologues represent a conserved “Ku domain” at the centre of the eukaryotic Ku complexes (amino acids 250–550 approximately; Figure 3) but lack other conserved domains present in the eukaryotic proteins [26,27]. The Ku domain of Ku70 and Ku80 is responsible for both heterodimerization and DNA binding, forming a ring-like structure through which the ends of the DNA break are threaded [22,30]. In contrast to the heterodimeric Ku complex of eukaryotes, the bacterial Ku complexes are predominantly homodimeric in structure, forming dimers that also bind preferentially to the ends of double-strand DNA [28] (Figure 3).

The Gam protein of bacteriophage Mu shares significant sequence and, probably, structural homology with both eukaryotic and prokaryotic Ku complexes [31]; (Figure 3). Gam orthologues are also present in a number of bacterial genomes, and these are believed to have derived from prophage insertions. In common with the prokaryotic Ku complexes, Gam is a homodimer that binds to DNA ends (Figure 3) and can protect linear Mu-phage DNA from nuclease digestion [31]. It has been proposed that Gam's role, following viral infection, is to bind to the ends of linear-phage DNA, preventing degradation by host exonucleases, and thus enhancing integration of the phage genome into the host's chromosome [31]. Interestingly, *E. coli* (strains expressing Gam, such as O157:H7) takes up linearized plasmid DNA much more efficiently than strains lacking this gene [32]. This strain, as well as other bacteria (e.g., *Neisseria meningitidis, Haemophilus influenzae*) possessing a phage-derived Gam, are naturally competent for DNA transformation. One of the major mechanisms for the evolution of bacteria involves the acquisition of genetic information by lateral gene transfer. Consequently, bacteria possessing Gam and other Ku homologues have the potential to acquire DNA directly from their surroundings and integrate this foreign genetic information into their genomes [31]. Therefore, it is possible that Ku may be instrumental in the process of prokaryotic evolution.

The similarity between the bacterial, phage, and eukaryotic Ku complexes suggests they have evolved from a common ancestral Ku, which functioned as a homodimeric complex that presumably bound to and protected the terminal ends of DNA (Figure 3). This primordial Ku most likely then evolved by merging with other functional domains to facilitate the recruitment of other factors, thus enhancing and diversifying the cellular role of Ku. Examples of probable domain acquisitions include the von Willebrant factor A (vWA) and SAP domains [26,27]. The vWA domain (Ku70/Ku80) enables Ku to recruit other proteins to sites of DNA damage, whilst the SAP motif (Ku70) binds to DNA and may prevent Ku from translocating away from the ends of DSBs [22]. The C-terminal end of *Streptomyces coelicolor* Ku protein shares significant homology with the SAP motif of Ku70 [26,27]. In effect, these apparent genetic acquisitions may have led to the evolution of an elementary NHEJ apparatus. Some bacterial genomes encode two distinct bacterial Ku genes within the same operon [26], possibly the result of an early gene-duplication event, giving rise to heterodimeric Ku complexes that are now the norm in eukaryotes.

**Prokaryotic Ku and ligase form a two-component NHEJ repair complex.** When the genome sequence for *Mycobacterium tuberculosis* H37Rv (Mt) was completed [33], it was immediately evident that this genome had the potential to encode four DNA ligases, referred to as Mt-LigA (NAD^+^-dependent ligase), Mt-LigB, Mt-LigC, and Mt-LigD (adenosine triphosphate [ATP]–dependent ligase). Although, at the time, this was thought to be rather unusual for a bacterial genome, many other prokaryotes are now known to harbour multiple DNA ligases [25,34]. Furthermore, bioinformatic analysis identified potential homologues of Ku in a similarly wide range of bacteria [26,27].

One striking observation, evident from the genetic order of the bacterial Ku genes, was that many of the Ku complexes are organized into operons containing ATP-dependent DNA ligases (homologues of Mt-LigD) [25–27]. It has now been established that this novel family of repair proteins are functional DNA ligases capable of catalyzing DSB rejoining in an ATP-dependent manner [28,29,35]. Eukaryotic Ku recruits DNA ligase IV to the termini of DSBs [16,17]. The circumstantial association of homologous genes in prokaryotic operons suggested that bacterial Ku complexes also recruit a repair ligase to DSBs [26,27]. Significantly, the end-joining activity of these ligases is specifically stimulated by the operon-associated Ku but not by eukaryotic Ku complexes, suggesting that together the Ku and ligase form a species-specific NHEJ complex [28]. Mutant strains of *Bacillus subtilis* bearing inactivating mutations in Ku *(YkoU)* and ligase *(YkoV)* homologues are sensitive to ionizing radiation in stationary phase, supporting the conclusion that Ku and ligase play a role in DSB repair in prokaryotes [28].

The majority of DSBs generated in vivo probably have ends that are non-complementary, and many breaks also have damaged termini. Therefore, the ends of these DSBs require processing by factors, including nucleases and DNA polymerases, to generate termini capable of being ligated (Figure 1). Eukaryotes produce many proteins that play a role in this processing [12,13,15]. In contrast, the prokaryotic NHEJ complexes appear to consist of only two repair factors (Ku and ligase) [25–27]. However, they have come up with a unique solution to compensate for the lack of additional processing factors. In addition to a ligase domain, many of the Ku-associated ligase genes encode domains with end-processing activities [25,27], including a polymerase that belongs to the Pol X family, members of which include human Pol μ, λ, and TdT, implicated in gap-filling during NHEJ. The Mt NHEJ repair ligase (Mt-LigD) possesses a remarkable variety of polymerase activities [29,36] including primase, terminal transferase, and gap-filling polymerase activities. A 3′ to 5′ single-strand DNA exonuclease activity also resides in the Mt-ligase polypeptide that is capable of removing 3′ overhangs [29,36]. Similar activities also reside in Pae-LigD, the homologous protein from *Pseudomonas aeruginosa* (37,38). Thus, many of the prokaryotic DNA-repair ligases are multi-functional DNA-repair enzymes that possess, with the exception of 5′-3′ exonuclease activity, all of the enzymatic activities required to process and join incompatible termini, irrespective of the ends. Indeed, DSB repair can be reconstituted in vitro simply by the addition of the Mt-Ku and ligase proteins.

Ku is not directly involved in the processing of breaks; however, it appears to be essential for recruiting ligase to the DSBs and may also modulate the order and extent of resection [29,36]. Mutations in yeast Ku and ligase genes can be complemented by ectopically expressing the bacterial NHEJ factors, confirming that reconstitution of NHEJ can also be established in vivo [28,29]. Together, these findings indicate that the prokaryotic NHEJ proteins constitute a fully functional two-component NHEJ repair complex.

**Figure 4:**
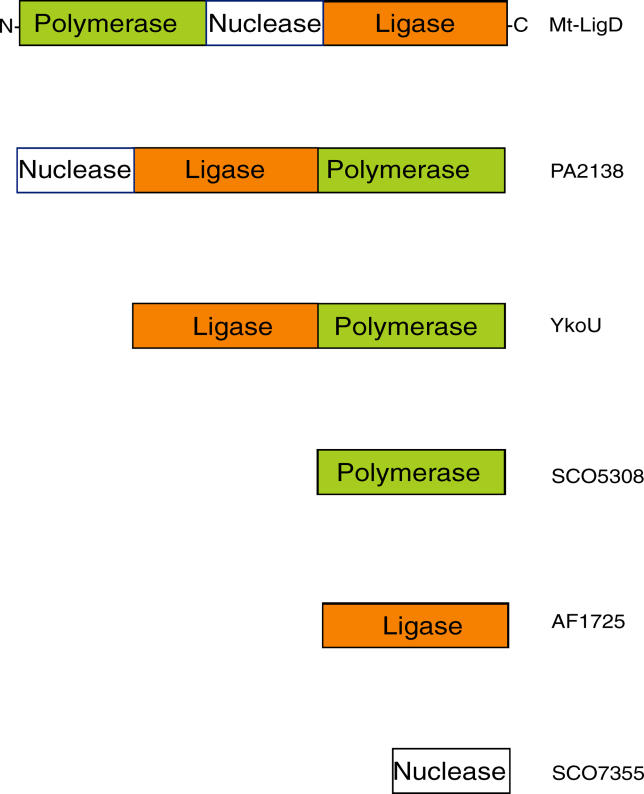
Domain Organization of Prokaryotic NHEJ Repair Enzymes Prokaryotic NHEJ DNA ligases exhibit a variety of arrangements of catalytic domains. Representative examples are presented showing the approximate location of the individual domains (amino acid): from Mt, Mt-LigD (polymerase, 1–280; nuclease, 281–460; ligase, 461–760); from *P. aeruginosa,* PA2138 (nuclease, 1–200; ligase, 201–550; polymerase, 551–841); and from *B. subtilis,* YkoU (ligase, 1–320; polymerase, 321–611). Putative “stand-alone” NHEJ proteins containing related DNA ligase, polymerase, and nuclease domains are also included—SCO refers to *S. coelicolor* and AF refers to *A. fulgidus.*

In the two best-studied examples of the end-joining ligases, Mt-LigD and Pae-LigD, the ancillary processing domains appear to be modular units [28,29,35–39]. Further analysis of genome sequences confirms this idea of distinct modules since homologous genes are organized in a variety of ways in different bacterial species (Figure 4). For example, in Mt*,* the polymerase and nuclease domains are distal and proximal amino-terminal extensions, respectively, of the ligase domain, and together they constitute a single polypeptide (Mt-LigD). The same activities can be found on a single polypeptide in *P. aeruginosa,* but the nuclease and polymerase domains are amino-terminal and carboxyl-terminal extensions, respectively, of the ligase domain. Furthermore, in *B. subtilis,* the polymerase domain resides at the C-terminal end of the ligase (YkoU), and the nuclease domain is absent [25,27]. Additional complexity is observed in other organisms, since the genome of *S. coelicolor* [26,27,40] encodes stand-alone genes for nuclease, polymerase, and ligase. Interestingly, Ku remains genetically associated with the separated genes for polymerase in *S. coelicolor* and ligase in *Archaeoglobus fulgidus* [26,27]. Thus, prokaryotes appear to have achieved a number of different solutions to providing a functional NHEJ pathway, although the manner in which these genes evolved is not yet clear. It also remains to be discovered whether or not the stand-alone ligase, nuclease, and polymerase proteins are capable of interacting in *trans,* reminiscent of eukaryotic NHEJ repair.

**Cellular roles of the NHEJ pathway in prokaryotic organisms.** Although it has been established that many bacterial species possess an NHEJ repair apparatus, the exact role and cell-cycle utilization of this pathway have yet to be elucidated. Many microorganisms that retain an NHEJ system spend much of their life-cycle in stationary phase, a stage of prolonged mitotic exit [41]. The transition from an actively dividing cell to a more quiescent cellular state is often a response to nutritional and environmental changes, and this transformation is accompanied by cellular adaptation, such as sporulation or microfilm formation. Prolonged cell-cycle exit has a number of important consequences for genome stability. During stationary phase, cells can be exposed for long periods of time to desiccation and other genotoxic agents that result in the accumulation of DSBs. Another consequence of stationary phase is that because there is a reduction in the average number of chromosomes per cell, it is expected that the ability of the cell to repair breaks via HR will be severely limited. Therefore it has been suggested that an NHEJ pathway may have evolved in bacteria to repair DSBs that arise at this particular stage of the cell-cycle [28,41].

Although deletion of the Ku and ligase in *B. subtilis* is not lethal, confirming that this pathway is non-essential for growth, the mutant strains do show mild sensitization to ionizing radiation in stationary phase [28]. Bacterial spores are highly resistant to irradiation and desiccation suggesting that they have an effective pathway(s) to repair resulting breaks or to prevent genome damage in the first place. As spores carry only one copy of their genome, it has been argued that an NHEJ pathway may be utilized to repair DSBs [42–44].

In some microorganisms, the onset of stationary phase results in the condensation of the chromosomal DNA into compact toroidal structures [42–45]. It has been argued that this morphology has a protective role and could also facilitate the repair of DSBs by limiting the diffusion of the termini of the breaks, thus enhancing the end-joining process, possibly by NHEJ [42–45]. A number of bacterial species that form these DNA structures, such as *Deinococcus radiodurans,* are capable of surviving very high levels of ionizing radiation (>15 kGy). This remarkable radio-resistance derives from the innate ability of these cells to repair hundreds of DSBs. Under conditions when chromosomal DNA is highly fragmented, it is likely to be more difficult to identify large regions of homologous DNA sequences, so HR is likely to be less effective. For example, the repair of DSB at the early stages following irradiation appears to be recA-independent [46], though ultimately, survival depends strongly on the recA HR protein.

Although Ku homologues have not been identified in *D. radiodurans,* arguing against the presence of NHEJ, a number of lines of evidence are emerging that may support the existence of an NHEJ-like pathway in this species. It has been reported that deletion of a unique radiation-inducible gene (PprA of *D. radiodurans*) leads to a significant sensitivity to ionizing radiation [47,48]. The PprA protein preferentially binds double-strand DNA carrying strand-breaks and stimulates DNA ligation, albeit in a non-specific way [48]. PprA could play a role similar to that of Ku although this remains to be established. It has also been reported that deletion of PolX_DR_, a member of the Pol X family, conferred sensitivity to irradiation, and this deletion strain also showed a significant delay in the restoration of genomic integrity following irradiation [49]. These reports suggest that *D. radiodurans,* and related bacteria, may possess a radiation-induced NHEJ apparatus that facilitates DSB repair, although the proteins that execute this repair process may be unique to these species.

Studies of mycobacteria have proved illuminating in respect of the in vivo function of the NHEJ proteins. Analysis of repaired junctions from individual NHEJ events confirmed that the DSBs are remodelled by the Mt end-joining complex [29,39]. Although LigD itself has the required activities to complete Ku-dependent NHEJ in mycobacteria, LigC may provide a back-up mechanism for LigD-independent repair of blunt-end DSBs [39]. Deletion strains of *mtligC* and *mtligD* are viable, so these gene products are not required for mycobacterial growth under laboratory conditions [35]. It is still possible that these genes influence the ability of the organism to infect their host cells. Indeed, a survey of deleted genes in 100 strains of clinically significant Mt did not find deletions in any of the ATP-dependent DNA ligases or in Mt-Ku [50]. In contrast, *M. leprae* has inactivated its ligase and Ku genes that could form an NHEJ apparatus, in accordance with the suggestions that this organism is downsizing its genome [51]. It has been speculated that the usefulness of NHEJ to mycobacteria is that it may allow repair of breaks induced by the genotoxic defence of human cells [39].

In addition, NHEJ may promote genetic diversity and, thus, some bacteria may have retained NHEJ since the genetic changes may provide growth advantages to bacteria under specific selection pressure, including the presence of antibiotics. NHEJ may represent a possible mechanism for generating such diversity via a process of stationary-phase mutagenesis that has been reported to occur in both prokaryotic and eukaryotic organisms [52–58]. Several molecular mechanisms have been proposed to explain the significant numbers of mutations that are produced during stationary phase and, indeed, various mechanisms may take place in different organisms or growth conditions [54–58]. There is evidence to implicate DNA damage in such types of mutation [52], but the roles of DNA-repair pathways in these processes have not been fully explored.

Current genomic-sequence data provide no obvious routes by which the prokaryotic NHEJ genes have been transferred between different organisms. Genomes of several rhizobia are now available [59] and provide perhaps the clearest indication that establishing evolutionary pathways for prokaryotic NHEJ will not be trivial. Homologues of Ku and the NHEJ ligase can be readily identified in *Mesorhizobium loti* and *Sinorhizobium meliloti* [25,26]. Using the Microbial Genome Database (http://mbgd.genome.ad.jp) [60], true homologues can also be identified in *Bradyrhizobium japonicum, Rhodopseudomonas palustris, Agrobacterium tumefaciens,* and *Nitrobacter hamburgensis* but are not present in species of Bartonella or brucella. This sporadic distribution of the genes suggests a complex phylogenetic relationship of NHEJ within the rhizobia (alpha-proteobacteria). A similar complex picture emerges when the other major phyla/classes of bacteria that contain NHEJ genes (β, γ, and δ proteobacteria, fimicutes, and actinobacteria) are considered in general (Figure 2). Although improvements in the number and depth of analysis of genome sequences might eventually alter these conclusions, the occurrence of these genes in bacteria is erratic. Given that the hosts are reasonably wide-ranging, it is not yet clear whether there are specific events or characteristics that promote acquisition and retention of the genes associated with NHEJ in prokaryotes. The most obvious explanation for this distribution is that the organisms acquired the NHEJ genes by horizontal gene transfer.

Perhaps the correlation of the possession of an NHEJ system is not determined by phylogeny but rather by the ecological environment of the bacterium. Indeed, it has been reported that among natural isolates of *E. coli,* the ability to engage in starvation/stress-induced mutagenesis is variable and is highly correlated with niche, and not with phylogeny [61]. Bjedov et al. showed that *E. coli* isolates from the gut of carnivores are more likely to be capable of stress-induced mutagenesis than isolates from the gut of herbivores [61]. This suggests that certain DNA metabolic activities, such as mutation and DNA repair, can evolve faster, depending on the niche, than the rest of the genome, which reflects phylogeny. Further studies are needed to determine if a similar environmental stress-driven evolutionary process accounts for the distribution of NHEJ genes in certain microorganisms and, if so, it may suggest what stresses (e.g., dessication, free-radical damage) promote this evolutionary process.

## Conclusions

As discussed within this review, experimental evidence for the existence of functional NHEJ genes/proteins has been obtained from three different types of bacteria: bacillus, mycobacteria, and pseudomonas. However, open reading frames that encode homologous proteins exist in the genomes of a wide range of diverse bacteria, though notably not in many of the organisms that have been well studied in the laboratory, such as *E. coli* K12. Recent studies with the prokaryotic proteins have already unearthed some unexpected and exciting observations. The diversity within the prokaryotic systems appears to offer the potential for many more interesting findings, including the discovery of the evolutionary relationships between the prokaryotic systems and also the evolutionary relationship with the homologous eukaryotic NHEJ pathway. It is apparent that the bacterial NHEJ apparatus represents an elegantly simple experimental model system that can be exploited to delineate the molecular mechanisms that coordinate the processing and joining of DSBs by NHEJ and also provide insights into the role of this pathway in bacterial cell processes. 

## Supporting Information

**Accession numbers** The GenBank (http://www.ncbi.nlm.nih.gov/Genbank) accession numbers for the genes and gene products discussed in this paper are Gam from bacteriophage Mu (70843), Mt-LigA (15610151), Mt-LigB (15610199), Mt-LigC (15610867), and Mt-LigD (6226918), the nuclease Artemis (13872809), Pae-LigD (15597334), the polymerases pol μ (29165858) and λ (57209464), PolX_DR_ (15805494), PprA (2257477), YkoU (2633694), and YkoV (2633695). The Swiss-Prot (http://www.ebi.ac.uk/swissprot) identification numbers for the *S. coelicolor* ligase, nuclease, and polymerase are SCO7345 (formerly SC4G10.24c), SCO7355 (formerly SC9H11.09c), and SCO5308 (formerly SC6G9.25), respectively, and for the Ku protein is SCO0601 (formerly SCF55.25c). The Swiss-Prot identification numbers for the Mt-LigD and and PolX_DR_ proteins are Rv0938 and DR0467, respectively.

## Abbreviations

ATP: adenosine triphosphate
DSB: DNA double-strand break
HR: homologous recombination
Mt: *Mycobacterium tuberculosis* H37Rv
NHEJ: non-homologous end-joining
vWA: von Willebrant factor A
